# Unsupervised gene function extraction using semantic vectors

**DOI:** 10.1093/database/bau084

**Published:** 2014-09-10

**Authors:** Ehsan Emadzadeh, Azadeh Nikfarjam, Rachel E. Ginn, Graciela Gonzalez

**Affiliations:** Department of Biomedical Informatics, Arizona State University, AZ 85259, USA

## Abstract

Finding gene functions discussed in the literature is an important task of information extraction (IE) from biomedical documents. Automated computational methodologies can significantly reduce the need for manual curation and improve quality of other related IE systems. We propose an open-IE method for the BioCreative IV GO shared task (subtask b), focused on finding gene function terms [Gene Ontology (GO) terms] for different genes in an article. The proposed open-IE approach is based on distributional semantic similarity over the GO terms. The method does not require annotated data for training, which makes it highly generalizable. We achieve an F-measure of 0.26 on the test-set in the official submission for BioCreative-GO shared task, the third highest F-measure among the seven participants in the shared task.

**Database URL**: https://code.google.com/p/rainbow-nlp/

## Introduction

Mining biomedical literature aims to reduce manual labor and provide enriched information that can empower advances in medical research and treatments. Lu *et al*. (1) demonstrated that there is an increasing interest to use text-mining techniques for curation workflows. Currently, literature curation is challenged by a lack of automated annotation techniques, particularly for Gene Ontology (GO) annotations (1). In medical informatics alone, the number of indexed articles has increased by an average of 12% each year between 1987 and 2006 (2, 3), with close to 20 million articles indexed in PubMed in 2013. With an increasing number of publications detailing complex information, the need to have reliable and generalizable computational techniques increases rapidly.

Finding gene functions discussed in literature is crucial to genomic information extraction (IE). Currently, tagging the gene functions in published literature is mainly a manual process. Curators find gene function evidence by reviewing each sentence in relevant articles and mapping the results to standard ontologies, and, specifically for this problem, to the GO (4), a controlled vocabulary of gene functions. The BioCreative IV GO workshop (5) aims to automate gene function curation through computational methods. With a focus on gene functions, it includes two subtasks: (i) retrieving GO evidence sentences for relevant genes and (ii) predicting GO terms for relevant genes. We focus on subtask b, which finds the related gene functions (GO terms) in a set of genes discussed in an article. More detail about the shared task and the corpus can be found in Auken *et al*. (6). This task is similar to BioCreative I subtask 2.2, which was held in 2004 (7). Blaschke *et al*. (7) summarized the results for BioCreative I. For subtask 2.2, the highest precision was reported to be 34.62% (8). BioCreative IV GO subtask 2 includes an annotated corpus to enable measurement of recall and F-measures. Couto *et al*. (9) used an information retrieval technique to find related sentences and GO terms. Chiang *et al*. (8) combined sentence classification with pattern mining. Ray *et al*. (10) proposed a solution based on probabilistic model and naïve Bayes classifier. Most of the participants in the previous related task focused on information content and statistical models combined with machine learning. Here, we propose an unsupervised method based on distributional semantic similarity that can be easily applied for different types of texts and ontologies.

We decided to apply an unsupervised method to see how well semantic similarity methods without any machine-learning or engineered features could perform compared with existing methods. If the performance is on par with the supervised methods, then we can benefit from unsupervised technique to leverage the performance of the supervised methods. In addition, finding gene functions discussed in a document seems a good application for semantic similarity, enabling comparison of different semantic similarity methods. None of the previous work in BioCreative I used semantic similarity methods, including vector- or graph-based methods. Our proposed technique is completely unsupervised, based solely on semantic similarity without training on the provided data set; this characteristic makes the method unlikely to overfit the data set and generalizable to the extraction of any major concepts mentioned in a document. The proposed method achieved the third highest F-measure among the seven participants in the shared task.

## Material and methods

Our method is based on distributional semantic similarity of sentences to GO terms. We use semantic vectors package (11) implementation of latent semantic analysis (LSA) (12) with random indexing (13) to calculate semantic similarities. GO terms’ semantic vectors are created based on GO names defined in GO; one semantic vector is created for each term in the ontology. Stop words are removed from GO name, and they are generalized by Porter stemming (14).

**Figure 1.**
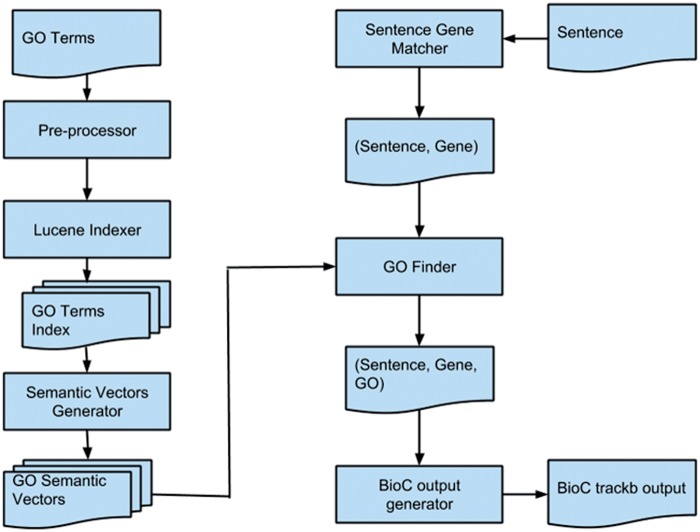
This diagram shows the high-level flow of the proposed system. The left column shows the steps to create semantic vectors for each GO term. The right column displays the steps for finding GO terms in a document.

Figure 1 shows the overall flow of our proposed method. After creating GO semantic vectors, the objective is to find whether a sentence is related to a gene. We do this by using lexical patterns and generalizing the sentence and gene symbol (e.g. removing the numbers and nonalphabetic characters). If ‘Sentence Gene Matcher’ predicts that a sentence is related to a gene, then we calculate its semantic similarity to all GO terms using already generated semantic vectors. The articles are provided in BioC format (15) in which sentences, passages and the types of passages (heading, paragraph, etc.) are identified. The ‘Go Finder’ module finds all related GO terms to the sentence and generates the triplet of sentence, gene and GO term. Finally, the shared task expected output format is generated by ‘BioC output generator’. In the next section, we explain the ‘GO Finder’ module in more detail.

### Semantic similarity

LSA with random indexing is used for calculating semantic similarity. LSA is a vector-based semantic similarity method that applies dimension reduction on document-term matrix before calculating cosine similarity of two terms.

The original proposed LSA algorithm uses singular value decomposition (SVD) for dimension reduction (12). This is a computationally expensive algorithm. Random indexing (13) technique has shown to be as effective as SVD but with linear complexity (16, 17). The semantic vectors are created for all GO terms regardless of their position in the GO graph. For creating document-word matrix, we consider each GO concept as a document and use the name field (‘GO term’) in GO for extracting terms of the node. A semantic vector is created for each concept in the ontology. For extracting terms from GO names, they are preprocessed by removing stop words (e.g. ‘the’, ‘a’), and then the extracted terms are generalized by Porter stemming (14).

### ‘GO Finder’ module

GO Finder finds related GO terms for each sentence. We define G as a set of top *m* GO terms with highest semantic similarity to the sentence. D is the set of top *n* GO terms with high similarity to the abstract of the related article. The following function returns top *k* similar GO terms for a given query:

$$\begin{array}{l} TopSimilarGO\left(query,\;k\right)\\ =\{x|x\in GOTerms\;\wedge \;|\{y\;\in GOTerms\;|\\ \;Sim(x,query)<Sim(y,query)\}|\;<k\} \end{array}$$

And G and D sets are

G(sentence)= TopSimilarGO(sentence,m)

D(abstract)= TopSimilarGO(abstract,n)

If a sentence is predicted to have the gene mention, the predicted GO terms for the sentence and gene are the conjunction of top similar GO terms to the sentence (set G) and top similar GO terms to the related abstract (set D):

$$\begin{array}{l} GeneGO(gene,sentence,abstract)\\ =\{G(sentence)\cap^{}D(abstract)\}ifHasGene\\ \left(sentence,gene)else\{\right\} \end{array}$$

A GO term with the highest semantic similarity to the sentence in the GeneGO set will be chosen as the final GO annotation for each gene in the sentence. For example, if a sentence top *m*( = 2) similar GO terms are {g5, g10} and the abstract top *n*( = 5) GO terms are {g4, g8, g5, g2, g9}, then the final predicted GO terms for the sentence related to the gene will be {g5}. The tuning parameters *m* and *n* control precision and recall.

**Figure 2.**
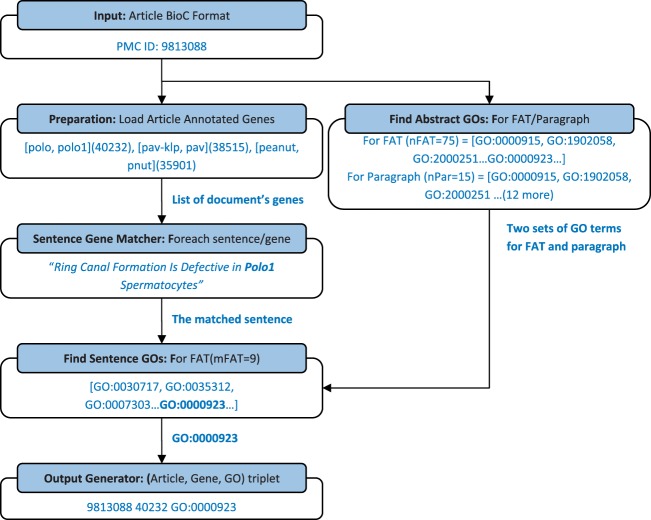
This flowchart shows the process of finding GO terms for each gene in a given document by an example. The example sentence category is ‘front_2’ (FAT sections). With the exception of the value for *n* and *m* parameters, the process is the same as FAT for sentences in paragraphs.

Table 1 summarizes the number of sentences in the training set that were detected by ‘Sentence Gene Matcher’ as relevant to a gene and also annotated to have a gene function. The table shows that ‘abstract’, ‘front’ and ‘title2’ sections of each document are the most important sections that can include gene function. The passage types appearing in Table 1 are taken exactly from the corpus. Table 2 shows an example for each passage types from publications in the train set. We found that the first sentences of paragraphs have information about GO terms, but including all sentences in a paragraph will significantly reduce the precision. Therefore, we limit searching for the gene functions to the mentioned sections of the article. We choose one set of values for *m* and *n*, for ‘**F**ront’, ‘**A**bstract’ and ‘**T**itle2’ (*mFAT, nFAT*), and choose a different set for the first sentence of the paragraphs (*mParagraph, nParagraph*). Figure 2 illustrates the process of generating output with an example. Next section shows detailed analysis of the impact of the tuning parameter on precision and recall.

**Table 1.** The table summarizes the number of sentences in the training set, which was detected by ‘Sentence Gene Matcher’ as relevant to a gene and also annotated to have a gene function

Passage type	With gene function	Total	%
front	26	67	39
title_2	149	797	19
abstract	225	1253	18
paragraph	1700	20 703	8
fig_title_caption	17	412	4
fig_caption	99	6009	2
table_title_caption	0	47	0
title_1, title_3, title_4	0	26	0

The different passage types are ‘front’ for the title of the article, ‘title_1’ refers to section headings like ‘Introduction’, ‘title_2’ is the section subheadings that sometimes describes the specific topic/finding of the section, ‘title_3’ and ‘title_4’ are more deeper levels of section headings, ‘abstract’ is the abstract content, ‘fig_title_caption’ is the title of a figure caption and ‘fig_caption’ is the caption of the figure, ‘table_title_caption’ is the caption of a table.

**Table 2.** This table lists description of different passage types appeared in the corpus along with an example for each type

Passage type	Description	Example
Front	The title of the document	Activation of ASK1, downstream MAPKK and MAPK isoforms during cardiac ischaemia
Abstract	The content of abstract section of the article	p38 MAPK is activated potently during cardiac ischaemia, although the precise mechanism by which it is activated is unclear. We used the isolated perfused rat heart …
Title_1	Section title	‘Introduction’, ‘Results’, ‘Discussion’
Title_2	Subsection title.	**Nuclear Translocation of Fussel through Medea**
Title_3	Subsubsection title. An inline heading that appears at the beginning of a paragraph.	**RNA interference by feeding**
		**GC analysis**
Title_4	An inline subheading that appears at the beginning of a paragraph.	**Materials**
		**Image Analysis**

Title_3 and Title_4 are similar, but we maintain the naming from the corpus to keep it consistent with the data.

### Evaluation method

The evaluation method is explained in detail by Mao *et al*. (5). The GO terms predicted by the system are compared with gold standard to calculate precision, recall and F-measure. In addition to exact match, hierarchical precision, recall and F-measure are used to evaluate the systems. In the hierarchical evaluation method, all of the ancestors of an annotated GO term in gold standard and system output are used to calculate the precision and recall. Hierarchical measures are calculated using the following formula below, where $Predicted_{expanded}$ and $Gold_{expanded}$ are system output and gold standard annotations expanded with the ancestor of selected GO terms in the ontology.

$$\begin{array}{l} P=\;\frac{\left|Predicted_{expanded}\cap^{\;}Gold_{expanded}\right|}{\left|Gold_{expanded}\right|},\;\\ R=\;\frac{\left|Predicted_{expanded}\cap^{}Gold_{expanded}\right|}{\left|Gold_{predicted}\right|} \end{array}$$

## Results and discussion

### Tuning parameters

**Figure 3.**
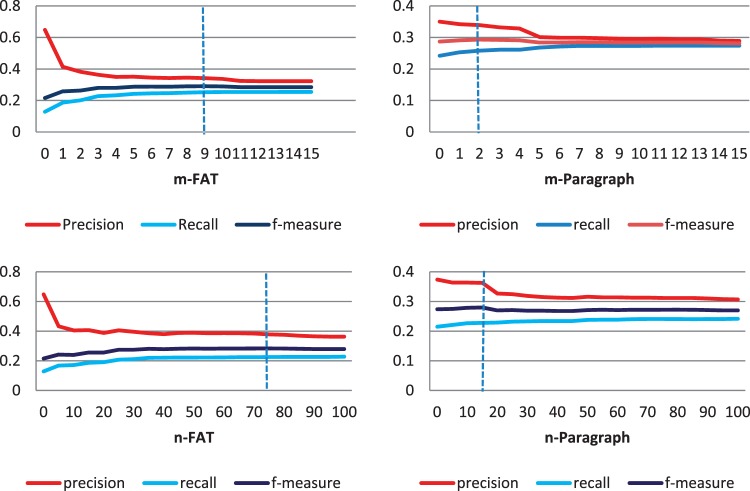
(a) Top-left diagram depicts precision, recall and F-measure change in respect to *mFAT* (‘Front’, ‘Abstract’ and ‘Title’) changes when other parameters have constant values (*mParagraph* = 1, *nFAT* = 100, *nParagraph* = 15). (**b**) Top-right diagram shows the change of performance based on changes of *mParagraph* when *mFAT* = 9, *nFAT* = 100, *nParagraph* = 15. (**c**) Bottom-left diagram shows the change of performance when *nFAT* varies and *mFAT* = 3, *mParagraph* = 1, *nParagraph* = 15. (**d**) Bottom-right diagram shows the change of performance when *nParagraph* varies and *mFAT* = 3, *mParagraph* = 1, *nFAT* = 100.

To achieve the highest F-measure, the tuning parameters (*m* and *n*) need to be adjusted accordingly. We use two sets of values for *m* and *n*; one set for the first sentence of each paragraph (*mParagraph* and *nParagraph*) and another for FAT passage types (*m_FAT_* and *nFAT*). To find the best tuning parameters, we evaluate the system with different values for a particular parameter while values of other parameters are constant. The experiment is repeated for all four parameters. Figure 3 shows variation of performance when tuning parameters change. Overall when parameters increase, precision increases and recall decreases. We tried to find the values that yield maximum F-measure. Figure 3a depicts precision, recall and F-measure change in respect to *mFAT* changes. As *mFAT* increases, precision declines and recall increases.

The maximum F-measure is achieved for *mFAT* = 9. Therefore, we assign *mFAT* to 9, and try to find the best value for *mParagraph*. Figure 3b shows the change of performance based on change of *mParagraph* and best result achieved for *mParagraph* = 15. Figure 3c shows variation of performance when *nFAT* varies, and Figure 3d shows performance change when *nParagraph* is changed while other parameters are constant. The best F-measure of 0.294 is achieved for *mFAT* = 9, *mParagraph* = 2, *nParagraph* = 15 and *nFAT* = 75*.*

When mParagraph varies, the change in F-measure is not as significant as when mFAT varies. In addition, recall is almost constant for mFAT >2. This shows that considering more than two GO terms for each sentence in FAT sections does not help us much and can only decrease the precision. On the other hand, considering only one top GO term for the first sentence of each paragraph gives the maximum boost to the recall.

### Results comparison

Having the tuned parameters, we compare the performance of the proposed intersection approach with alternative systems (without intersection algorithm or limit on section types). In addition, we compare the contribution of the first and the last sentences of paragraphs. Table 3 shows the performance of different settings. The first experiment tests how much the intersection approach improves the results in comparison to just finding semantic similarity of each sentence. The first four rows in Table 3 do not use intersection and simply use the most similar GO term to each sentence. The last five rows in Table 3 use the intersection method. The best recall (0.518) is achieved by not using intersection and not limiting scope to any specific part of the document; however, the precision is low.

**Table 3.** This table shows performance of different settings on dev-set

	Precision	Recall	F-measure
No intersection/All sections included	0.082	**0.518**	0.141
No intersection/Paragraph+FAT	0.091	0.498	0.155
No intersection/Paragraph	0.092	0.493	0.155
No intersection/FAT	0.281	0.272	0.276
Intersection/All section	0.268	0.305	0.285
Intersection/Paragraph last sentence+FAT	0.346	0.245	0.287
Intersection/Paragraph all sentences+FAT	0.316	0.278	0.296
Intersection/Paragraph last and first sentences+FAT	0.348	0.261	**0.299**
Intersection/Paragraph first sentence+FAT	**0.366**	0.252	0.298

For intersection approach, the tuning parameter values are *mFAT* = 9, *mParagraph* = 2, *nParagraph* = 15 and *nFAT* = 75. Random index algorithm random function’s seed was fixed to ‘1234’.

Limiting the scope to paragraph and FAT improved the precision slightly (+0.009) and decreased recall (−0.020). Similarly, including only Paragraph section improved precision and reduced recall a little (+0.010 precision, −0.025 recall). When only the FAT section is included, the precision increased significantly and recall also dropped sharply (+0.199 precision, −0.246 recall). This yields a higher F-measure than including paragraph or all sections. In short, when we limit the scope, the precision increases and recall decreases. We see the same pattern with intersection approach, but precision remains high in comparison with no-intersection approach. When we compare intersection and no-intersection approaches including all sections (Table 3, row 1 and row 5), it shows that intersection reduces recall by 0.213 but increases the precision by 0.186. In another experiment, we found that limiting search to first sentence of paragraph sections can improve the precision significantly. The last four rows of Table 3 compare the performance when different parts of the paragraph are included; they show that including the first sentence yields the best F-measure and precision.

In Table 4, we compared four settings for creating semantic vectors: (i) using only the GO terms, (ii) using GO term and definition, (iii) using GO term and synonym and (iv) using GO term, definition and synonym. Using only terms to create vectors achieves the best results. This may be mainly to the similarity of GO terms, and more description inclusion causes the vector to easily return incorrect GO term with higher similarity.

**Table 4.** Four settings for creating semantic vectors are compared in this table: (i) using only the GO terms, (ii) using GO term and definition, (iii) using GO term and synonym and (iv) using GO term, definition and synonym. For all experiments in this table, FAT and Paragraph (only first sentence) sections are considered

	Precision	Recall	F-measure
Create vectors with GO terms only	**0.366**	0.252	**0.298**
Create vectors with GO terms+definitions	0.247	0.229	0.238
Create vectors with GO terms+definitions+ synonyms	0.227	0.196	0.210
Create vectors with GO terms+synonym	0.197	0.189	0.193

## Conclusion

We proposed an unsupervised approach to extract gene functions from documents. The proposed approach only uses GO terms’ names for creating semantic vectors. We tried using GO terms description, but it does not help. Using a more fine-tuned vocabulary set for each GO term may result in more accurate vectors and may increase the performance of this method. In addition, using term–term semantic similarity for expanding sentence terms can be evaluated. We used annotations for finding the important passage types, evaluating the method and finding the best settings for the parameters. The main advantage of using unsupervised open-IE technique is that it can easily be generalized and applied to similar relation extraction problems. The results from this method can be used as a baseline for supervised systems. In the future, we plan to combine this approach with supervised techniques. The source code and outputs of each experiment are available in https://code.google.com/p/rainbow-nlp/.

## Funding

Research reported in this publication was supported by the National Library of Medicine under Award Number R01LM011176. The content is solely the responsibility of the authors and does not necessarily represent the official views of the National Library of Medicine. Funding for open access charge: National Library of Medicine, Award Number R01LM011176.

*Conflict of interest*. None declared.
